# Genome-wide analysis of *TMEM38* family revealed functional roles of *TMEM38B* in fat deposition and its miRNA-mediated regulation in chicken

**DOI:** 10.1016/j.psj.2025.105694

**Published:** 2025-08-16

**Authors:** Shuohan Li, Xi Cheng, Ke Zhang, Yang Wang, Hongyu Wei, Yihao Zhi, Zhimin Cheng, Yulong Guo, Hong Li, Yadong Tian, Xiaojun Liu, Weihua Tian

**Affiliations:** a College of Animal Science and Technology, Henan Agricultural University, Zhengzhou 450046, China; b Henan Key Laboratory for Innovation and Utilization of Chicken Germplasm Resources, Zhengzhou 450046, China; c International Joint Research Laboratory for Poultry Breeding of Henan, Zhengzhou 450046, China

**Keywords:** *TMEM38B*, miR-20b-3p, Abdominal fat deposition, Intramuscular fat deposition, Chicken

## Abstract

Transmembrane protein 38 (***TMEM38***) gene family, including *TMEM38A* and *TMEM38B*, is responsible for facilitating trimeric intracellular cation transport across the membrane and regulating key cellular processes, such as muscle contraction and cell differentiation in mammals. However, a genome-wide analysis of the chicken *TMEM38* gene family, as well as investigations into their biological roles and post-transcriptional expression regulation in fat deposition have not yet been conducted. In this study, we investigated the genome-wide characteristics of chicken *TMEM38* gene family, elucidated the regulatory roles of the *TMEM38B* gene in both abdominal and intramuscular adipogenesis, and explored its miRNA-mediated expression regulatory mechanisms. We found that chicken *TMEM38A* and *TMEM38B* exhibited notable conservation in gene structure and motifs across diverse species. Principal component analysis based on SNPs showed that genetic variations in the *TMEM38B* gene contributed to the selective breeding of commercial broilers. Moreover, gene expression profiling demonstrated that *TMEM38A* and *TMEM38B* showed the positive expression in chicken abdominal adipose and muscle tissues, and overall increased expression during the proliferation and adipogenic differentiation of both chicken abdominal and intramuscular preadipocytes. Functionally, *TMEM38B* overexpression significantly enhanced viability, proliferation, cell cycle progression as well as intracellular triglyceride content and lipid droplet accumulation of both chicken abdominal and intramuscular preadipocytes, paralleling with the expression of proliferative and adipogenic marker genes. Target miRNA prediction identified 37 potential miRNAs targeting the *TMEM38B* gene. Of these, a dual-luciferase reporter system verified that miR-20b-3p could directly bind to the 3′UTR of the *TMEM38B* gene and thus inhibit its post-transcriptional expression. Gain-of-function assays showed that miR-20b-3p could suppress the viability, proliferation, and cell cycle progression of chicken abdominal and intramuscular preadipocytes, as well as the adipogenic differentiation of chicken abdominal preadipocytes. Collectively, we demonstrated the promotive effects of *TMEM38B* in regulating abdominal and intramuscular fat deposition, as well as its post-transcriptional expression inhibition mediated by miR-20b-3p. These findings shed novel lights into the functional role and expression regulation of the miR-20b-3p-*TMEM38B* axis in adipogenesis, and may provide valuable biomarkers for the genetic improvement of fat traits in chickens.

## Introduction

Over the past few decades, intensive selection for growth rate, feed conversion efficiency, and lean meat yield has greatly enhanced production efficiency, but has been accompanied by excessive abdominal fat deposition and declined meat quality. Fat consists of glycerol and fatty acids, which play essential physiological roles in energy storage and thermoregulation (Wang et al., 2013). Abdominal fat (**AbF**) is considered a byproduct of slaughter, and its excessive deposition in chickens negatively affects feed conversion efficiency, egg production, and slaughter performance (Wang et al., 2017). Intramuscular fat (**IMF**) is considered a key indicator influencing meat quality, including flavor and tenderness, and is primarily distributed between the perimysium, endomysium, and epimysium. Both AbF and IMF deposition are influenced by environmental factors, nutritional composition and genetic regulation, with genetics being the most effective approach for selection and breeding (Abdalla et al., 2018; Tan and Jiang, 2024; Nematbakhsh et al., 2021; Xing et al., 2025). Therefore, it is of great significance to comprehensively explore fat-regulatory genes and elucidate their mechanisms for both fundamental research and molecular breeding strategies.

AbF and IMF mainly expand through the proliferation (numbers) of preadipocytes and their adipogenic differentiation (preadipocytes mature into lipid-filled adipocytes), termed as adipogenesis, which is a well-orchestrated processes involving various transcriptional and post-transcriptional mechanisms (Gregoire et al., 1998; Lefterova and Lazar, 2009). In chickens, a cascade of regulatory factors, including non-coding RNAs (ncRNAs) and protein-coding genes underlying AbF and IMF deposition have been validated by multi-omics technology and functional validation, respectively (Cao et al., 2023; Wang et al., 2024a; Xing et al., 2025; Zhai et al., 2023). However, little is known about the molecules responsible for the coordinated regulation of chicken AbF and IMF deposition.

The transmembrane protein 38 (*TMEM38*) gene family (*TMEM38A* and *TMEM38B*) encodes TRimeric Intracellular Cation channel subtypes (**TRIC**), which facilitate trimeric intracellular cation transport across membranes and regulate endoplasmic reticulum (**ER**) calcium homeostasis, thereby influencing various mammalian cellular processes including muscle function. *TMEM38A* could regulate calcium transport in cardiomyocytes by modulating calcium ion signaling (Zhou et al., 2021). Its absence in mice led to hypertension and induces abnormal calcium handling in skeletal muscle (Yamazaki et al., 2011; Zhao et al., 2010). Additionally, *TMEM38A* was reported to promote apoptosis in renal cancer cell lines, such as 786-O and Caki-1 (Zhao et al., 2025). Furthermore, TRIC-A encoded by *TMEM38A* gene was expressed during the early developmental stages of zebrafish, suggesting its potential role in development (Tonelli et al., 2023). *TMEM38B* knockdown could disrupt calcium signaling, thereby affecting skeletal development, collagen metabolism, and osteogenic differentiation (Cabral et al., 2016; Jovanovic et al., 2023). Mutations in *TMEM38B* gene were associated with autosomal recessive osteogenesis imperfecta, which manifests clinically as bone deformities, as well as muscle and cardiac dysfunction (Jovanovic and Marini, 2024). Moreover, our previous analysis of Hi-C, ATAC-seq and RNA-seq in breast muscle from commercial fast-growing Arbor Acres (**AA**) broilers and slow-growing Yufen (**YF**) indigenous chickens suggested that a YF-specific gain-accessible region linked to *TMEM38B* gene resulted in higher *TMEM38B* expression, which may promote IMF deposition in YF chickens (Wang et al., 2024b). However, the specific functional roles and expression regulation of *TMEM38B* gene in fat deposition remain undefined.

As a subclass of ncRNAs, microRNAs (**miRNAs**) are pivotal post-transcriptional regulators that modulate gene expression by interacting with the 3′ untranslated regions (**UTRs**) of target genes to trigger mRNA degradation and translational repression, thus participating in various processes such as cell proliferation, differentiation, apoptosis, inflammation, and metabolism (Bartel, 2009; Hart et al., 2024; Peng and Croce, 2016). In chickens, several miRNAs have been identified as key regulators of the proliferation and adipogenic differentiation of chicken abdominal and intramuscular preadipocytes, including miR-24-3p (Lin et al., 2022), let-7a-3p (Ren et al., 2024), miR-128-3p (Zhu et al., 2023), miR-2188-3p (Guo et al., 2023), miR-27b-3p (Zhang et al., 2019), and miR-30d-5p (Chen et al., 2023) for chicken intramuscular preadipocytes; as well as miR-106-5p (Tian et al., 2022), miR-429-3p (Chao et al., 2020), miR-125b-5p (Li et al., 2021), miR-301b-3p (Tian et al., 2023), miR-125-5p (Li et al., 2021), miR-17-92 (Zhang et al., 2017), miR-24-3p (Guo et al., 2021), and miR-122-5p (Zhai et al., 2023) for chicken abdominal preadipocytes. However, no miRNA has been found to simultaneously regulate both abdominal and intramuscular adipogenesis.

In this study, we systematically analyzed the phylogenetic characteristics, gene structures, protein physicochemical properties, genetic variation patterns and expression patterns of the chicken *TMEM38* gene family, assessed the regulatory roles of the *TMEM38B* gene in chicken abdominal and intramuscular adipogenesis based on preadipocyte proliferation and differentiation models, and investigated the miRNA-mediated post-transcriptional regulation of *TMEM38B* expression and molecular mechanisms underlying adipogenesis. To the best knowledge, this is the first study to identify the coordinated regulation of the miRNA/target-gene axis in chicken AbF and IMF deposition. Our study not only offers new insights into coordinately regulatory mechanisms of fat deposition, but also provides theoretical foundations and potential genetic targets for the improvement of fat traits in chickens.

## Materials and methods

### Ethical statement

This study was approved by the Animal Care and Use Committee of Henan Agricultural University (Approval No. 11-0085). All procedures involving experimental chickens were conducted in strict accordance with the "Guidelines for the Humane Killing of Experimental Animals" (T/CALAS 31-2017) issued by the Chinese Association for Laboratory Animal Science.

### Identification, gene structure, protein physicochemical properties and expression patterns of chicken TMEM38 gene family

To identify *TMEM38* family members in chickens, a Hidden Markov Model (**HMM**) was constructed based on the amino acid sequences of known human TMEM38 proteins using HMMER 3.0 with default parameters (Johnson et al., 2010). This HMM was then applied to identify putative TMEM38 family members in the GRCg7b.protein.faa provided by the National Center for Biotechnology Information (**NCBI**). Gene annotation files (**GFF3 format**) for all chicken *TMEM38* genes were also obtained from Ensembl, and their exon-intron structures were visualized using TBtools (Chen et al., 2020). The molecular weight (**MW**), theoretical isoelectric point (**pI**), instability index (**II**), and grand average of hydropathicity (**GRAVY**) index for each TMEM38 protein were calculated using ExPASy online tools. The tissue expression patterns were obtained from the Tissue Gene Expression Atlas of Domesticated Chickens developed by the Wang Lab (https://chickenatlas.avianscu.com/) (Zhang et al., 2022).

### Evolutionary characteristics, domain, and conserved motif analysis of chicken TMEM38 gene family

TMEM38 family protein sequences were collected from eight representative species: human (*Homo sapiens*), mouse (*Mus musculus*), chicken (*Gallus gallus*), quail (*Coturnix japonica*), Western clawed frog (*Xenopus tropicalis*), Chinese softshell turtle (*Pelodiscus sinensis*), Viviparous lizard (*Zootoca vivipara*), and channel catfish (*Ictalurus punctatus*). Multiple sequence alignment was performed using the MUSCLE algorithm implemented in MEGA11 software with default parameters. A phylogenetic tree was constructed using the Neighbor-Joining (**NJ**) method with 1,000 bootstrap replicates to ensure robustness. Conserved protein domains were predicted using the Batch CD-Search tool, while conserved motifs were identified using MEME software, setting the number of motifs to 15.

### Principal component analysis of genetic variations in chicken TMEM38 gene family

To investigate the genetic variation characteristics of the chicken *TMEM38* gene family, principal component analysis (**PCA**) was performed using genome resequencing data from ten chicken breeds, including five indigenous breeds (Lushi, Gushi, Xichuan Black, Zhengyang Sanhuang, and Henan Local Chicken; 10 samples per breed), two commercial broiler breeds (Cobb broiler and Cryptic White broiler; 30 samples per breed), two commercial egg-laying breeds (White Leghorn and Lohmann Brown; 10 samples per breed), and one wild-type breed (Red Jungle Fowl; 5 samples). Single nucleotide polymorphisms (SNPs) located in the 5 kb upstream regulatory regions, 5′ UTRs, exons, introns, and 3′ UTRs of the *TMEM38A* and *TMEM38B* genes were extracted for PCA to assess genetic structure and domestication patterns among the chicken populations.

### Bioinformatics analysis of chicken TMEM38B gene

Signal peptides were predicted using SignalP 6.0 (https://services.healthtech.dtu.dk/). Physicochemical properties were analyzed using ProtParam (https://web.expasy.org/protparam/). Transmembrane domains were identified using TMHMM 2.0 (https://services.healthtech.dtu.dk/), and subcellular localization was predicted using WoLF PSORT (https://www.genscript.com/tools/wolf-psort). Online tools, including miRDB (http://mirdb.org/miRDB/), microT-CDS (http://diana.imis.athena-innovation.gr/DianaTools/index.php?r=microT_CDS/), and TargetScan (http://www.targetscan.org/vert_72/), were utilized to predict miRNAs which potentially interact with the 3′ UTR of the *TMEM38B* gene. RNAhybrid was subsequently used to analyze the duplex formation and minimum free energy (**MFE**) of miRNA and *TMEM38B* 3′ UTR duplexes.

### Cell isolation and culture

Chicken intramuscular preadipocytes were isolated from the breast muscle of 14-day-old AA broilers as previously described by Zhang et al. (2020). The cells were cultured in complete medium composed of DMEM/F12 (Gibco, Gaithersburg, MD, USA), 10 % fetal bovine serum (**FBS**; Gibco, Gaithersburg, MD, USA), and 1 % penicillin-streptomycin (Gibco, Gaithersburg, MD, USA). Immortalized chicken preadipocytes 2 (**ICP2**) cells were obtained from the Key Laboratory of Chicken Genetics and Breeding (Northeast Agricultural University, Harbin, Heilongjiang Province, China) and cultured in DMEM complete medium consisting of DMEM (Gibco, Gaithersburg, MD, USA), 10 % FBS (Gibco, Gaithersburg, MD, USA), and 1 % penicillin-streptomycin (Solarbio, Beijing, China). Chicken embryonic fibroblast DF-1 cells were obtained from the American Type Culture Collection (**ATCC**) (Manassas, USA) and maintained in DMEM (Gibco, Gaithersburg, MD, USA) complete medium. All cells were maintained at 37 °C in a humidified incubator with 5 % CO₂. Upon reaching 90 % confluence, adipogenic differentiation of chicken intramuscular preadipocytes and ICP2 cells was induced by treating the cells with a differentiation medium composed of complete medium supplemented with 160 μM oleic acid (Solarbio, Beijing, China) dissolved in dimethyl sulfoxide (Solarbio, Beijing, China).

### RNA extraction, cDNA synthesis, and real-time quantitative PCR (qPCR)

Total RNA was extracted using the TRIzol reagent (Vazyme Biotech, Nanjing, China) according to the manufacturer's instructions, and first-strand cDNA was synthesized using the HiScript Reverse Transcription Kit (Vazyme Biotech, Nanjing, China). The qPCR was conducted on a Roche LightCycler 96 system in a 10 μL reaction volume containing 0.4 μL each of forward and reverse primers (10 μM), 1 μL (approximately 100 ng) of cDNA, 3.2 μL of nuclease-free water, and 5 μL 2 × ChamQ Universal SYBR qPCR Master Mix (Vazyme Biotech, Nanjing, China). The qPCR was performed using the SYBR Green method on a LightCycler 96 system (Roche, Basel, Switzerland). The qPCR protocol consisted of an initial denaturation at 95 °C for 5 minutes, followed by 40 cycles of denaturation at 95 °C for 30 seconds, annealing at 60 °C for 30 seconds, and extension at 72 °C for 20 seconds. Dissociation curve analysis was then conducted with an initial step at 95 °C for 10 s, annealing at 65 °C for 20 s, and a gradual increase in temperature to 97 °C with data acquisition every 0.2 s. The cDNA templates were serially diluted to generate standard curves, and primer efficiencies were calculated (E = [10^(-1/slope) - 1] × 100 %). Only primer pairs with 90 %-110 % efficiencies and a single dissociation curve were used for qPCR analysis. *β-actin* served as the internal reference gene to normalize mRNA expression levels. All reactions were performed in triplicate. The qPCR primers (Supplementary Table S1) were designed using Primer Premier 5.0 software and synthesized by Qingke Biotechnology Co., Ltd. Relative gene expression was calculated using the 2^−ΔΔCT^ method.

### Plasmid construction and cell transfection

To construct *TMEM38B* gene overexpression plasmid, the coding sequence (**CDS**) of *TMEM38B* gene was amplified by PCR and inserted into the pcDNA3.1-EGFP vector (Invitrogen, Carlsbad, CA, USA) using HindIII and EcoRI restriction sites, named as pcDNA3.1-TMEM38B-EGFP. Transfection was carried out using Lipofectamine 3000 reagent (Invitrogen, Carlsbad, CA, USA) according to the manufacturer’s protocol. miR-20b-3p mimics (sense: ACUGUAAUGUGGGCACUUACAG, anti-sense: GUAAGUGCCCACAUUACAGUUU) and the miR-20b-3p mimics negative control (NC) (sense: UUCUCCGAACGUGUCACGUTT, anti-sense: ACGUGACACGUUCGGAGAATT) were synthesized by GenePharma Co., Ltd (Shanghai, China).

To verify whether miR-20b-3p targets the *TMEM38B* gene, *TMEM38B* 3′UTR containing and lacking the miR-20b-3p binding site were amplified and inserted into XhoI and NotI restriction enzymes (Takara, Kyoto, Japan) double-digested psi-CHECK™-2 plasmid (Promega, Madison, WI, USA), respectively, designated as TMEM38B-3′UTR-WT (wild-type plasmid) and TMEM38B-3′UTR-mut (mutant plasmid).

### Triglyceride (TG) content measurement

Cells (3 × 10⁴ cells/well) were seeded into 12-well plates and cultured in complete medium. Following adipogenic induction, cells were washed three times with 1 × phosphate-buffered saline (**PBS**) (Solarbio, Beijing, China) and lysed using lysis buffer. Intracellular TG content was quantified using a Cellular Triglyceride Content Assay Kit (Applygen, Beijing, China) in accordance with the manufacturer’s instructions. Total protein concentration was measured using a BCA protein assay kit (Epizyme, Shanghai, China) to normalize TG content.

### Oil Red O staining and quantification

Following adipogenic induction, cells were washed three times with 1 × PBS, fixed with 4 % paraformaldehyde, and stained with a 40 % Oil Red O solution (Sigma, St. Louis, MO, USA). Lipid droplets accumulation was visualized using a microscope (Olympus, Tokyo, Japan). For quantification, intracellular lipid droplets were extracted with 100 % isopropanol (Sigma, St. Louis, MO, USA), and measured at 500 nm absorbance.

### CCK-8 assay

Cells (1 × 10³ cells/well) were seeded into 96-well plates and cultured in complete medium. At 12, 24, 36, and 48 h post-transfection, cell proliferation was evaluated using a CCK-8 Cell Counting Kit (Dojindo, Kumamoto, Japan) according to the manufacturer’s instructions. After incubating with 10 μL CCK-8 reagent in the dark for 2 h, absorbance was measured at 450 nm using a microplate reader (BioTek, Winooski, VT, USA). Each group included 12 replicates. To account for background absorbance in the cells, the blank control (containing only the medium and CCK-8 reagent without cells) absorbance were subtracted from the experimental absorbance.

### 5-Ethynyl‐2‐deoxyuridine (EdU) assay

Cells (3 × 10⁴ cells/well) were seeded into 12-well plates and cultured in complete medium. At 24 h post-transfection, cells were incubated with EdU reagent (diluted 1:1000 in complete medium) at 37 °C in a humidified incubator with 5 % CO₂ for 2 h using an EdU assay kit (Ribobio, Guangzhou, China) following the manufacturer’s protocol. EdU-positive cells were stained red. Cell nuclei were stained blue with Hoechst 33342 (Ribobio, Guangzhou, China). Fluorescence images were captured using a fluorescent inverted microscope (Olympus, Tokyo, Japan).

### Flow cytometry analysis

Cells (3 × 10⁵ cells/well) were seeded into 6-well plates and cultured in complete medium. At 24 h post-transfection, cells were harvested, washed three times with 1 × PBS, and fixed in 75 % ethanol. Cell cycle analysis was performed using a BD AccuriC6 flow cytometer (BD Biosciences, San Diego, CA, USA) using the Cell Cycle Detection Kit (KeyGEN Biotech, Nanjing, China) in accordance with the manufacturer’s instructions.

### Dual-luciferase reporter assay

To validate the interaction between miR-20b-3p and *TMEM38B* gene, DF1 cells were seeded into 24-well plates and co-transfected with 300 ng of either wild-type or mutant reporter plasmid and 50 nM miR-20b-3p mimics or negative control (**NC**) using Lipofectamine™ 3000 (Thermo Fisher Scientific, Waltham, MA, USA). After 48 h, cells were washed three times with 1 × PBS and lysed in passive lysis buffer. Firefly luciferase and *Renilla* luciferase activity was measured using the Dual-Luciferase® Reporter Assay System (Promega, Madison, WI, USA) on a microplate reader. *Renilla* luciferase activity was normalized to firefly luciferase activity.

### Statistical analysis

Data are presented as mean ± standard error of the mean (**SEM**). Independent sample t-test was used for comparisons between two groups, while one-way analysis of variance (**ANOVA**) and Duncan’s multiple range test was applied for comparisons among three or more groups using SPSS 24.0 (IBM, Chicago, IL, USA). Statistical significance was defined as *P < 0.05, extremely significant differences as **P < 0.01, and non-significant differences as *P* > 0.05. All graphical representations were generated using GraphPad Prism 8.0 (GraphPad Software, San Diego, CA, USA).

## Results

### Genome-wide identification, characteristics, genetic variations and expression profiles of TMEM38 gene family

**Fig. 1:**
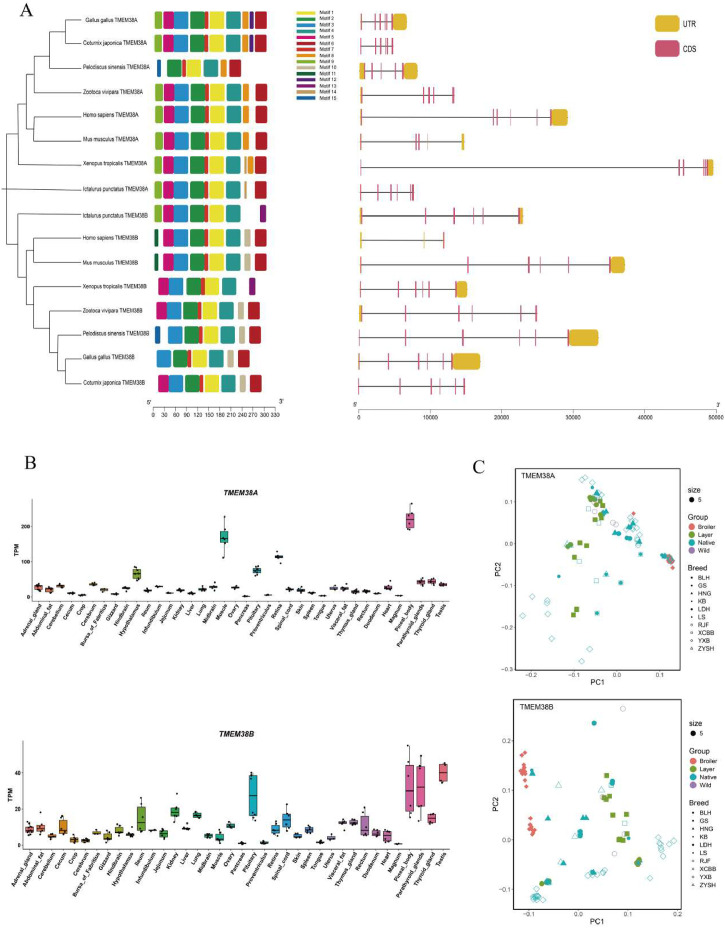
Genome-wide characteristics of *TMEM38* gene family. (A) Analysis of evolutionary features, conserved motifs and gene structure of TMEM38 family. (B) Tissue expression profile of chicken TMEM38A and TMEM38B genes. (C) Principal component analysis of single nucleotide polymorphisms in TMEM38A and TMEM38B genes across multiple chicken breeds.

Based on the amino acid sequences of human TMEM38 family members, two TMEM38 subfamily members—*TMEM38A* and *TMEM38B*—were identified in chickens, located on chromosome 28 and chromosome Z, respectively. The protein physicochemical properties of chicken TMEM38A and TMEM38B were characterized in Supplementary Table S2, and both were characterized as hydrophobic and unstable proteins. The phylogenetic tree of the TMEM38 family across different species reveals two distinct subgroups (TMEM38A and TMEM38B), with both avian (chicken and quail) TMEM38A and TMEM38B clustering into a branch with the Chinese softshell turtle. TMEM38A and TMEM38B within the same subgroup shared similar gene structures and conserved motifs (Fig. 1A).

Tissue expression profiling showed that *TMEM38A* and *TMEM38B* were positively expressed in chicken abdominal adipose and muscle tissues; however, their expression levels in abdominal adipose tissue were lower than some other tissues, such as lung, pituitary, ovary (Fig. 1B). To investigate the response of the *TMEM38A* and *TMEM38B* genes to domestication among wild, indigenous and commercialized chicken breeds, respectively, a total of 107 and 236 SNPs within 5 kb promoters, 5′UTRs, exons, introns and 3′ UTRs were screened among multiple chicken breeds (Supplementary Table S3). The results of PCA revealed significant genetic variation in the *TMEM38B* gene between commercial and wild or indigenous breeds, suggesting that *TMEM38B* played a significant role in selective breeding of commercial broilers. In contrast, the *TMEM38A* gene showed no significant differentiation between commercial and indigenous or wild breeds, indicating that the *TMEM38A* gene did not respond to artificial selection (Fig. 1C). Based on these results, chicken *TMEM38B* was selected for further functional validation. Protein function prediction analysis indicated that chicken *TMEM38B* did not possess a signal peptide (Supplementary Fig. S1A), contained a characteristic transmembrane domain, and was classified as a membrane protein (Supplementary Fig. S1B). Subcellular localization analysis showed that chicken *TMEM38B* was predominantly localized to the endoplasmic reticulum, indicating its potential role in lipid synthesis and lipid droplet formation (Supplementary Fig. S1C). These results indicate that chicken *TMEM38B* is a transmembrane protein localized to the endoplasmic reticulum and may exert regulatory functions in adipogenesis.

### Effect of TMEM38B on proliferation of chicken intramuscular preadipocytes

**Fig. 2:**
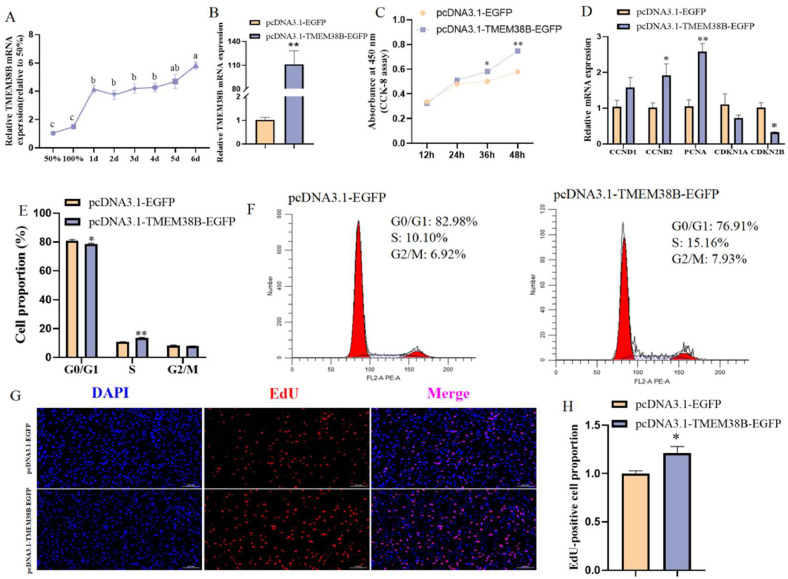
Effect of *TMEM38B* gene on chicken intramuscular preadipocytes proliferation. (A) Relative *TMEM38B* mRNA expression during intramuscular preadipocytes proliferation and differentiation. Different lowercase letters indicate significant differences, while the same lowercase letters indicate no significant differences. The same below. (B) Detection of TMEM38B overexpression efficiency. (C) CCK-8 assay for the proliferation of chicken intramuscular preadipocytes upon *TMEM38B* overexpression. (D) Effects of TMEM38B overexpression on the expression of proliferation-related marker genes. (E) Flow cytometry assay for cell cycle analysis of chicken intramuscular preadipocytes upon *TMEM38B* overexpression. (F) Representative flow cytometry diagram for cell cycle analysis. (G) EdU staining for the proliferation of chicken intramuscular preadipocytes upon *TMEM38B* overexpression. (H) Quantification of the number of EdU-positive cells. *P < 0.05,^⁎⁎^P < 0.01.

The qRT-PCR analysis showed that the mRNA expression levels of *TMEM38B* gradually increased during the proliferation and adipogenic differentiation stages of chicken intramuscular preadipocytes (Fig. 2A), suggesting a regulatory role in chicken intramuscular adipogenesis. To investigate the functions of *TMEM38B* in proliferation of chicken intramuscular preadipocytes, cells were transfected with either pcDNA3.1-EGFP (control) or pcDNA3.1-*TMEM38B*-EGFP (overexpression) plasmids. Compared to control cells, *TMEM38B* mRNA expression was significantly elevated (approximately 110-fold) in cells transfected with the *TMEM38B* overexpression plasmid (Fig. 2B). CCK-8 assays revealed that *TMEM38B* overexpression significantly enhanced cell viability of chicken intramuscular preadipocytes at 36 h and 48 h post-transfection (Fig. 2C). Additionally, overexpression of *TMEM38B* significantly upregulated the mRNA expression of cell proliferation-promoting marker genes (*CCNB2* and *PCNA*), while significantly downregulated that of cell proliferation-inhibition marker gene (*CDKN2B*) (Fig. 2D). Flow cytometry analysis showed that *TMEM38B* overexpression facilitated cell cycle progression from the G0/G1 phase to the S phase, resulting in a marked decrease in the proportion of G0/G1 phase cells and an increase in that of S phase cells (Fig. 2E and F). Additionally, EdU staining assay demonstrated that *TMEM38B* overexpression significantly increased the proportion of EdU-positive cells (Fig. 2G and H). Collectively, these results indicate that *TMEM38B* promotes the proliferation of chicken intramuscular preadipocytes.

### Effect of TMEM38B on adipogeneic differentiation of chicken intramuscular preadipocytes

**Fig. 3:**
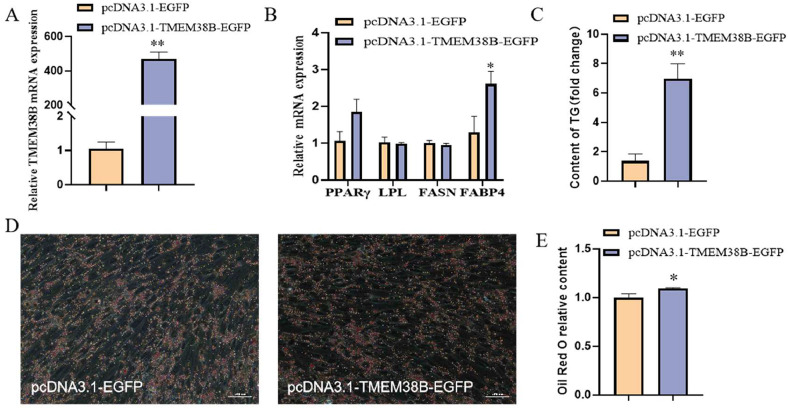
Effect of *TMEM38B* gene on chicken intramuscular preadipocytes adipogenic differentiation. (A) Detection of TMEM38B overexpression efficiency. (B) The relative mRNA expression of PPARγ,LPL,FASN and FABP4 in differentiated chicken intramuscular preadipocytes upon TMEM38B overexpression. (C) Detection of intracellular TG content. (D) Detection of intracellular lipid droplet accumulation by Oil Red O staining. (E) Spectrophotometric analysis of intracellular lipid droplet content. *P < 0.05,^⁎⁎^P < 0.01.

To investigate the function of *TMEM38B* in adipogenic differentiation of chicken intramuscular preadipocytes, cells were transfected with either pcDNA3.1-EGFP (control) or pcDNA3.1-*TMEM38B*-EGFP (overexpression) plasmids, and subsequently induced to undergo adipogenic differentiation. Compared to control cells, *TMEM38B* mRNA expression was significantly elevated (approximately 400-fold) in cells transfected with the pcDNA3.1-*TMEM38B*-EGFP plasmid (Fig. 3A). Consistently, *TMEM38B* overexpression significantly upregulated the mRNA expression of the lipid synthesis-related *FABP4* gene (Fig. 3B). Furthermore, intracellular lipid droplet accumulation and TG content were markedly increased in *TMEM38B*-overexpressing cells (Fig. 3C–E). Taken together, these findings suggest that *TMEM38B* overexpression promotes adipogenic differentiation of chicken intramuscular preadipocytes.

### Effect of TMEM38B on proliferation of chicken abdominal preadipocytes

**Fig. 4:**
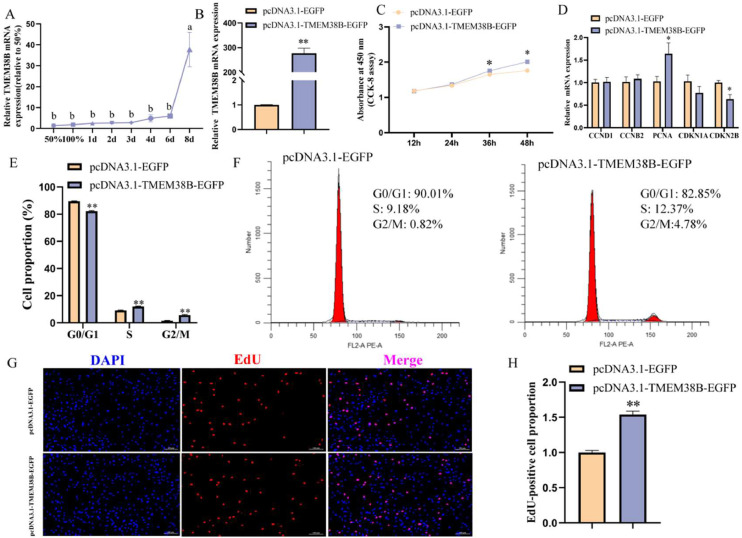
Effect of T*MEM38B* gene on chicken abdominal preadipocytes proliferation. (A) Relative *TMEM38B* mRNA expression during chicken abdominal preadipocytes proliferation and differentiation. (B) Detection of TMEM38B overexpression efficiency. (C) CCK-8 assay for the proliferation of chicken abdominal preadipocytes upon TMEM38B overexpression. (D) Effects of TMEM38B overexpression on the expression of proliferation-related marker genes. (E) Flow cytometry assay for cell cycle of chicken abdominal preadipocytes upon TMEM38B overexpression. (F) Representative flow cytometry diagram for cell cycle analysis. (G) EdU staining for the proliferation of chicken abdominal preadipocytes upon TMEM38B overexpression. (H) Quantification of the number of EdU-positive cells. *P < 0.05,^⁎⁎^P < 0.01.

As determined by qRT-PCR analysis, *TMEM38B* showed an overall increased mRNA expression during the proliferation and adipogenic differentiation stages of chicken abdominal preadipocytes (ICP2 cells) (Fig. 4A), indicating a potential regulatory role in chicken abdominal adipogenesis. To investigate the function of *TMEM38B* in the proliferation of chicken abdominal preadipocytes, ICP2 cells were transfected with either the pcDNA3.1-EGFP control plasmid or the pcDNA3.1-*TMEM38B*-EGFP overexpression plasmid. Compared to control cells, *TMEM38B* mRNA expression was significantly elevated (approximately 280-fold) in the *TMEM38B* overexpressing cells (Fig. 4B). CCK-8 assays demonstrated that *TMEM38B* overexpression significantly enhanced cell viability at 36 h and 48 h post-transfection (Fig. 4C). In addition, *TMEM38B* overexpression significantly upregulated the mRNA expression of the cell proliferation-promoting marker gene *PCNA* and downregulated that of the cell proliferation-inhibition marker gene *CDKN2B* (Fig. 4D). Flow cytometry analysis further showed that overexpression of *TMEM38B* promoted cell cycle progression from G0/G1 phase to S phase and G2/M phase, resulting in a marked decrease in the proportion of G0/G1 phase cells and increase in that of S phase cells (Fig. 4E and F). Consistently, EdU staining assay showed that *TMEM38B* overexpression significantly enhanced the proportion of EdU-positive ICP2 cells (Fig. 4G and H). Collectively, these results indicate that *TMEM38B* overexpression promotes the proliferation of chicken abdominal preadipocytes.

### Effect of TMEM38B on adipogenic differentiation of chicken abdominal preadipocytes

**Fig. 5:**
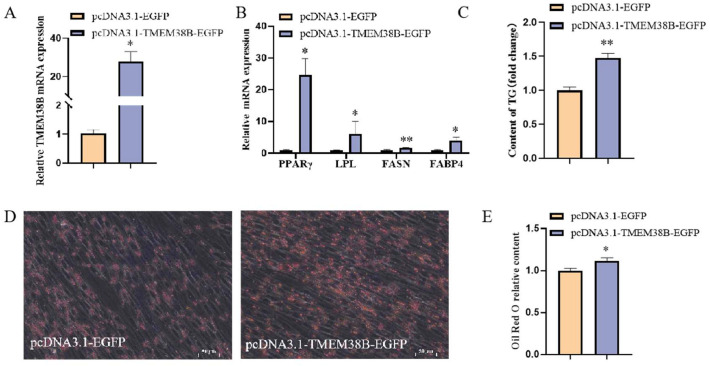
Effect of *TMEM38B* gene on chicken abdominal preadipocytes adipogenic differentiation. (A) Detection of TMEM38B overexpression efficiency. (B) The relative mRNA expression of PPARγ,LPL,FASN and FABP4 in differentiated chicken abdominal preadipocytes upon TMEM38B overexpression. (C) Detection of intracellular TG content. (D) Detection of intracellular lipid droplet accumulation by Oil Red O staining. (E) Spectrophotometric analysis of intracellular lipid droplet content. *P < 0.05,^⁎⁎^P < 0.01.

To investigate the function of *TMEM38B* in the adipogenic differentiation of chicken abdominal preadipocytes, ICP2 cells were induced to undergo adipogenic differentiation, followed by transfection with either pcDNA3.1-EGFP (control) or pcDNA3.1-*TMEM38B*-EGFP (overexpression) plasmids. Compared to control cells, *TMEM38B* mRNA expression was significantly elevated (approximately 28-fold) in cells transfected with the pcDNA3.1-*TMEM38B*-EGFP plasmid (Fig. 5A). Moreover, *TMEM38B* overexpression significantly upregulated the mRNA expression of key adipogenic marker genes, including *PPARγ, LPL, FASN*, and *FABP4* (Fig. 5B), which was accompanied by a marked increase in lipid droplet accumulation and intracellular TG content in differentiated ICP2 cells (Fig. 5C–E). Collectively, these results indicate that *TMEM38B* promotes adipogenic differentiation of chicken abdominal preadipocytes.

### Screening and validation of miRNAs targeting TMEM38B gene

**Fig. 6:**
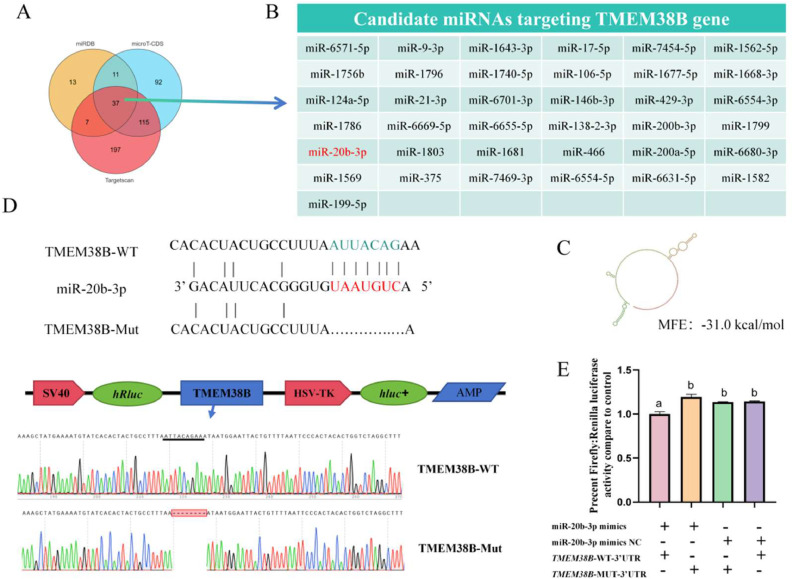
Validation of the *TMEM38B* gene as a direct target of miR-20b-3p. (A) Prediction of the miRNAs targeting *TMEM38B* gene by Targetscan, microT-CDS and miRDB software. (B) Candidate miRNAs targeting *TMEM38B* gene. (C) Secondary structure of the RNA duplex of miR-20b-3p and the 3′ UTR of *TMEM38B* gene. Red indicates the target 3′ UTR of *TMEM38B* gene; green indicates miR-20b-3p. (D) Complementary sequences of miR-20b-3p and the 3′ UTR of *TMEM38B* gene. (E) validation of the interaction between miR-20b-3p and TMEM38B 3′ UTR by a dual-luciferase reporter assay. *p < 0.05, **p < 0.01.

To identify miRNAs implicated in adipogenesis that target chicken *TMEM38B* gene, we firstly integrated predictions from Targetscan, miRDB and microT-CDS tools to predict the miRNAs which directly bind to the 3′UTR of *TMEM38B* gene. A total of 37 miRNAs were screened as candidates targeting the *TMEM38B* gene (Fig. 6A–B, Supplementary Table S3). Of these, miR-20b-3p showed an overall decrease expression during the adipogenic differentiation of ICP2 cells based on previous RNA-seq data (Tian et al., 2021), which showed a significant negative correlation with *TMEM38B* mRNA expression (Supplementary Fig. S2A and B). The minimum free energy (**MFE**) of the miRNA–mRNA duplex was calculated as −31.0 kcal/mol, indicating strong hybridization stability (Fig. 6C). miR-20b-3p was therefore selected as a candidate for miRNA–mRNA target validation. To validate the interaction between miR-20b-3p and the *TMEM38B* gene, we successfully constucted the wild-type and mutant plasmids that contained and lacked the complementary binding site (the 2–8 nt seed region of miR-20b-3p and the 496–162 nt region of the *TMEM38B* 3′ UTR), respectively (Fig. 6D). The wild-type or mutant plasmids were co-transfected with miR-20b-3p mimics or mimic negative control (NC) into DF1 cells. Dual-luciferase reporter assay results demonstrated that miR-20b-3p significantly suppressed the relative luciferase activity of the wild-type plasmid (*TMEM38B*-3′UTR-WT), but had no significant effect on the mutant plasmid (*TMEM38B*-3′UTR-mut) (Fig. 6E). These findings confirm that the *TMEM38B* gene is a direct target of miR-20b-3p.

### Effect of miR-20b-3p on chicken intramuscular preadipocyte proliferation

**Fig. 7:**
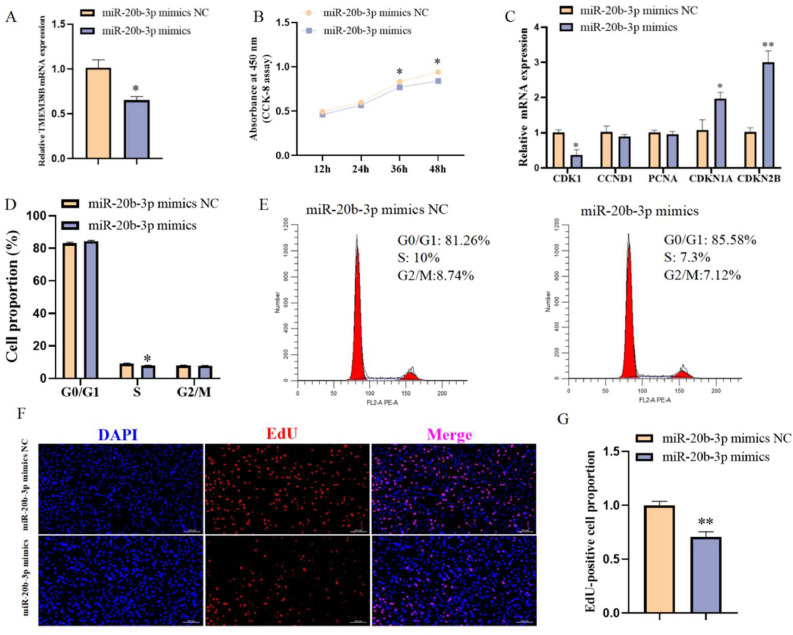
Effect of miR-20b-3p on chicken intramuscular preadipocyte proliferation. (A) qRT-PCR analysis of miR-20b-3p expression in chicken intramuscular preadipocytes transfected with miR-20b-3p mimics and miR-20b-3p mimics NC. (B) Cell viability of chicken intramuscular preadipocytes transfected with miR-20b-3p mimics and miR-20b-3p mimics NC by CCK-8 assay. (C) Relative mRNA expression of *CDK1, CCND1, PCNA, CDKN1A* and *CDKN2B* in chicken intramuscular preadipocytes transfected with miR-20b-3p mimics and miR-20b-3p mimics NC. (D) Cell cycle analysis of chicken intramuscular preadipocytes transfected with miR-20b-3p mimics and miR-20b-3p mimics NC. (E) Representative flow cytometry diagram of cell cycle analysis. (F) EdU staining of chicken intramuscular preadipocytes transfected with miR-20b-3p mimics and miR-20b-3p mimics NC. (G) Quantification of the number of EdU-positive cells. *P < 0.05,^⁎⁎^P < 0.01.

To investigate the role of miR-20b-3p in the proliferation of chicken intramuscular preadipocytes, miR-20b-3p was overexpressed by transfecting cells with either miR-20b-3p mimics or mimic negative control (NC). Following miR-20b-3p overexpression, the mRNA expression of its target *TMEM38B* gene was significantly reduced (Fig. 7A). CCK-8 assays demonstrated that miR-20b-3p overexpression significantly suppressed cell viability at 36h and 48 h post-transfection (Fig. 7B). Moreover, miR-20b-3p mimics significantly downregulated cell proliferation-promotion marker gene *CDK1* mRNA expression while upregulating cell proliferation-inhibition marker gene *CDKN1A* and *CDKN2B* mRNA expression (Fig. 7C). Flow cytometry analysis revealed that miR-20b-3p overexpression resulted in a marked reduction in the proportion of S phase cells (Fig. 7D and E). Consistently, EdU staining assay showed that miR-20b-3p overexpression significantly reduced the proportion of EdU-positive chicken intramuscular preadipocytes (Fig. 7F and G). Collectively, these results indicate that miR-20b-3p inhibits the proliferation of chicken intramuscular preadipocytes.

### Effects of miR-20b-3p on adipogeneic differentiation of chicken intramuscular preadipocytes

**Fig. 8:**
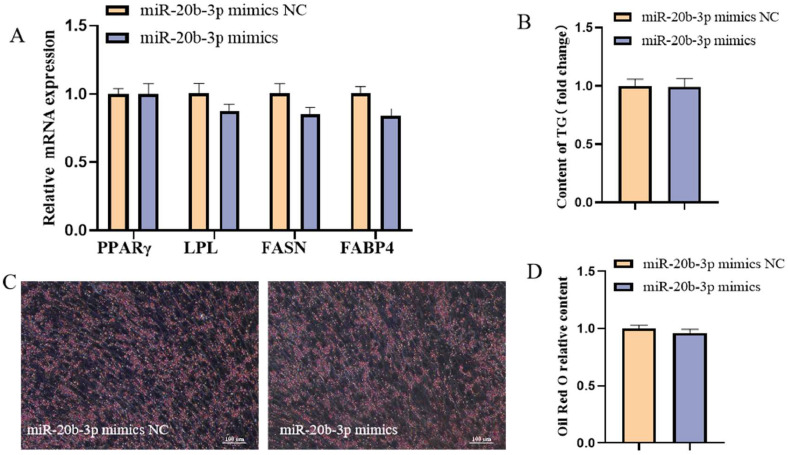
Effect of miR-20b-3p on adipogeneic differentiation of chicken intramuscular preadipocytes. (A) The relative mRNA expression of PPARγ, LPL, FASN and FABP4 in differentiated chicken intramuscular preadipocytes transfected with miR-20b-3p mimics and miR-20b-3p mimics NC. (B) Detection of intracellular TG content. (C) Oil red O staining. (D) Spectrophotometric analysis of intracellular lipid droplet content.

To investigate the effects of miR-20b-3p on the adipogenic differentiation of chicken intramuscular preadipocytes, cells were induced to undergo adipogenic differentiation, followed by transfection with either miR-20b-3p mimics or mimic negative control (**NC**). As determined by qRT-PCR analysis, compared to the control cells, the expression levels of key adipogenic marker genes—*PPARγ, LPL, FASN*, and *FABP4*—were not significantly altered following miR-20b-3p overexpression (Fig. 8A). In addition, no significant differences were observed in lipid droplet accumulation or intracellular TG content (Fig. 8B–D). Collectively, these findings indicate that miR-20b-3p does not significantly influence the adipogenic differentiation of chicken intramuscular preadipocytes.

### Effects of miR-20b-3p on chicken abdominal preadipocyte proliferation

**Fig. 9:**
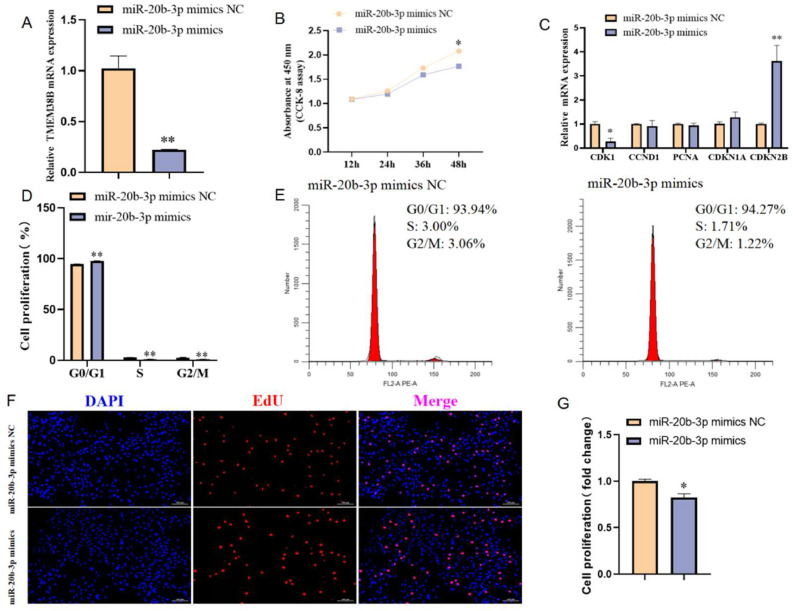
Effect of miR-20b-3p on chicken abdominal preadipocyte proliferation. (A) qRT-PCR analysis of miR-20b-3p expression in chicken abdominal preadipocytes transfected with miR-20b-3p mimics and miR-20b-3p mimics NC. (B) Cell viability of chicken abdominal preadipocytes transfected with miR-20b-3p mimics and miR-20b-3p mimics NC by CCK-8 assay. (C) Relative mRNA expression of *CDK1, CCND1, PCNA, CDKN1A* and *CDKN2B* in chicken abdominal preadipocytes transfected with miR-20b-3p mimics and miR-20b-3p mimics NC. (D) Cell cycle analysis of chicken abdominal preadipocytes transfected with miR-20b-3p mimics and miR-20b-3p mimics NC. (E) Representative flow cytometry diagram of cell cycle analysis. (F) EdU staining of chicken abdominal preadipocytes transfected with miR-20b-3p mimics and miR-20b-3p mimics NC. (G) Quantification of the number of EdU-positive cells. *P < 0.05,^⁎⁎^P < 0.01.

To investigate the functional role of miR-20b-3p in chicken abdominal preadipocyte proliferation, miR-20b-3p was overexpressed in ICP2 cells by transfection with either miR-20b-3p mimics or mimic NC. Following miR-20b-3p overexpression, the mRNA expression of its target gene, *TMEM38B*, was significantly reduced (Fig. 9A). CCK-8 assay indicated that miR-20b-3p overexpression significantly suppressed cell viability at 48 h post-transfection (Fig. 9B). In addition, miR-20b-3p mimics significantly downregulated cell proliferation-promotion marker gene *CDK1* mRNA expression while upregulating cell proliferation-inhibition marker gene *CDKN2B* mRNA expression (Fig. 9C). Flow cytometry analysis further showed that overexpression of miR-20b-3p inhibited cell cycle progression from G0/G1 phase to S phase and G2/M phase, resulting in a marked increase in the proportion of G0/G1 phase cells and a decrease in the proportion of S phase and G2/M phase cells (Fig. 9D and E). EdU staining assays further demonstrated that miR-20b-3p overexpression significantly reduced the proportion of EdU-positive ICP2 cells (Fig. 9F and G). Taken together, these findings suggest that miR-20b-3p inhibits the proliferation of chicken abdominal preadipocytes.

### Effects of miR-20b-3p on adipogenic differentiation of chicken abdominal preadipocytes

**Fig. 10:**
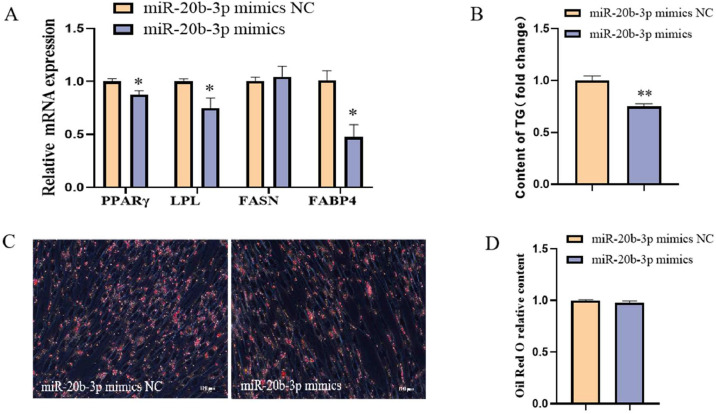
Effect of miR-20b-3p on adipogeneic differentiation of chicken abdominal preadipocytes. (A) The relative mRNA expression of PPARγ, LPL, FASN and FABP4 in differentiated chicken abdominal preadipocytes transfected with miR-20b-3p mimics and miR-20b-3p mimics NC. (B) Detection of intracellular TG content. (C) Oil red O staining. (D) Spectrophotometric analysis of intracellular lipid droplet content. *P < 0.05,^⁎⁎^P < 0.01.

To examine the role of miR-20b-3p in the adipogenic differentiation of chicken abdominal preadipocytes, ICP2 cells were induced to undergo adipogenic differentiation, followed by transfection with either miR-20b-3p mimics or mimic NC. The qRT-PCR analysis revealed that miR-20b-3p overexpression significantly downregulated the expression of key adipogenic markers, including *PPARγ, LPL*, and *FABP4* (Fig. 10A). Compared to control cells, there were no significant differences in lipid droplet accumulation, whereas intracellular TG content was significantly reduced in ICP2 cells treated with miR-20b-3p mimics (Fig. 10B–D). Collectively, these findings suggest that miR-20b-3p inhibits the adipogenic differentiation of chicken abdominal preadipocytes.

## Discussion

Fat deposition in poultry production not only directly influences meat quality but also represents a critical trait requiring precise regulation in genetic improvement and breeding programs. In this study, we focused on the miR-20b-3p/*TMEM38B* axis and systematically investigated its regulatory mechanisms governing cell proliferation and adipogenic differentiation in chicken preadipocytes, including both abdominal and intramuscular preadipocytes. These findings provide new insights into the molecular network underlying fat deposition in poultry.

*TMEM38B* belongs to the *TMEM38* gene family, which encodes TRIC channels that function in intracellular calcium homeostasis and bone development (Jovanovic et al., 2023). The TRIC family consists of two members: TRIC-A (encoded by *TMEM38A*) and TRIC-B (encoded by *TMEM38B*). TRIC-A and TRIC-B are homologous trimers with similar biochemical functions, but they differ in their regulatory mechanisms and expression patterns. TRIC-A is predominantly expressed in excitable cells, such as muscle, where it is localized to the sarcoplasmic reticulum membrane, whereas TRIC-B is localized in the endoplasmic reticulum membranes of various cell types and tissues (Yazawa et al., 2007). Our phylogenetic tree of the TMEM38 family across different species reveals two subgroups, *TMEM38A* and *TMEM38B*, with each subgroup displaying conserved motifs. Long-term artificial selection has generated remarkable phenotypic differences among indigenous chicken breeds, commercial broilers, commercial layers, and wild chicken breeds. PCA analysis based on SNPs of chicken *TMEM38* gene family showed that the indigenous chicken breeds, commercial broilers, commercial layers, and wild red jungle fowl populations could be clearly separated by *TMEM38B* gene and could not be clearly separated by *TMEM38A* gene, indicating that the genetic variations of *TMEM38B* gene, not *TMEM38A* gene, contributed to the domestication and formation of the specialized traits in the modern commercial broilers. A possible explanation is that the *TMEM38A* expression pattern may be more conserved or genetically constrained, making it less prone to accumulate beneficial mutations. In contrast, *TMEM38B* has undergone stronger positive selection during commercial broiler breeding and might influence muscle development.

It was reported that Tmem38b-deficient mice exhibited a variety of dramatic phenotypes, including ER swelling, impaired collagen release, and poor bone ossification (Zhao et al., 2016). Phenotypic observations in humans and mice suggest that disorders such as sclerosing osteogenesis imperfecta (excessively thick bones) and phospholipidosis (excessive accumulation of phospholipids) can be ameliorated by inhibition of TRIC-B (Hu and Gulyaeva, 2019). It has been increasingly revealed that calcium signaling pathways such as CAMK, MAPK/ERK, and NFAT regulate cell cycle progression and lipid metabolism (López-Victorio et al., 2013; Zhai et al., 2020; Zhang et al., 2018). In the present study, *TMEM38B* gene expression showed an overall increase during the proliferation and adipogenic differentiation of both chicken intramuscular and abdominal preadipocytes, indicating that *TMEM38B* participates in chicken intramuscular and abdominal adipogenesis. Further in vitro study demonstrated that *TMEM38B* promoted the proliferation, cell cycle progression, and adipogenic differentiation of both chicken intramuscular and abdominal preadipocytes, indicating its promotive roles in these processes.

Generally, miRNAs interacts with the 3′ UTR of its target genes to post-transcriptionally silence mRNA expression, thus participating in various physiological processes. In this study, *TMEM38B* gene was validated as a direct target of miR-20b-3p, which could directly bind to the 3′ UTR of *TMEM38B* gene and suppress its mRNA expression. miR-20b-3p, a member of the miR-17 family, has been implicated in metabolic disorders, neural damage, and pathological processes such as diabetic neuropathy, autophagy, and tumorigenesis (Bartel, 2018; Khalilian et al., 2022; Li et al., 2023; Lu et al., 2020; Shahani et al., 2025). However, its regulatory role in avian adipogenesis remains inadequately characterized and needs systematic investigation. In this study, miR-20b-3p overexpression resulted in a conspicuous decrease in the proliferation and cell cycle progression of chicken intramuscular preadipocytes, and simultaneously led to a remarkable decrease in the proliferation, cell cycle progression and adipogenic differentiation of chicken abdominal preadipocytes. This indicates that miR-20b-3p serve as an inhibitor of both chicken intramuscular and abdominal adipogenesis.

Despite robust molecular and cellular evidence supporting the role of the miR-20b-3p/TMEM38B axis in fat deposition, several avenues remain for future research. For example, although *TMEM38B* acts as an activator of proliferation, cell cycle progression and adipogenic differentiation in both chicken intramuscular and abdominal preadipocytes, its precise regulatory mechanisms remain unclear. *TMEM38B* can function as a monovalent cation channel and affect calcium homeostasis in the endoplasmic reticulum, thereby regulating calcium signaling pathways such as CAMK, MAPK/ERK, and NFAT, that govern cell cycle progression and lipid metabolism (López-Victorio et al., 2013; Zhai et al., 2020; Zhang et al., 2018). Therefore, we speculate that chicken *TMEM38B* might regulate calcium homeostasis in chicken intramuscular and abdominal preadipocytes and thus affect their proliferation, cell cycle progression and adipogenic differentiation, which needs further investigation. Furthermore, although the synchronously inhibition effects of miR-20b-3p in the proliferation and cell cycle progression of both chicken intramuscular and abdominal preadipocytes, miR-20b-3p inhibited the adipogenic differentiation of chicken abdominal preadipocytes but did not affect that of chicken intramuscular preadipocytes, namely tissue-specific regulatory pattern of miR-20b-3p underlying fat deposition in chickens. It was generally accepted that miRNA has multiple target genes and can simultaneously regulate their expression. Besides *TMEM38B* gene, miR-20b-3p can target the other genes responsible for adipogensis in chicken intramuscular preadipocytes, thus ultimately counteracting the accelerating effects of *TMEM38* gene in their adipogenic differentiation and this may be attributable to differences in transcription factor pools, signaling pathway activation, or epigenetic states, providing important directions for future mechanistic studies.

## Conclusion

**Fig. 11:**
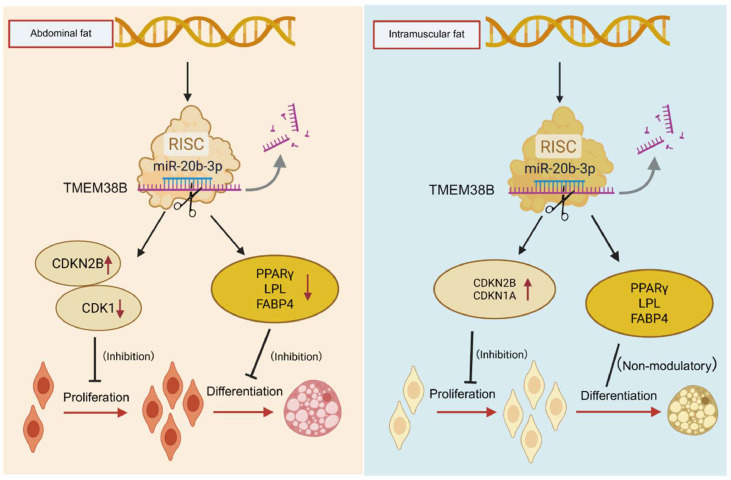
Schematic illustration of miR-20b-3p regulating fat deposition through targeted modulation of *TMEM38B* gene in chickens.

In summary, this study demonstrated for the first time genome-wide identification, characteristics and genetic variations of chicken *TMEM38* gene family, and found *TMEM38B* gene was responded to the divergent selection for broilers. The in vitro assay revealed the promotion effects of *TMEM38* gene in the proliferation, cell cycle progression and adipogeneic differentiation of both chicken intramuscular and abdominal preadipocytes. Moreover, *TMEM38B* gene was directly targeted by miR-20b-3p to post-transcriptionally inhibit its mRNA expression. miR-20b-3p could inhibit the proliferation, cell cycle progression and adipogeneic differentiation of chicken abdominal preadipocytes as well as the proliferation and cell cycle progression of chicken intramuscular preadipocytes (Fig. 11). The miR-20b-3p/*TMEM38B* axis regulating the fat deposition not only sheds light on deeper understanding of molecular mechanisms underlying fat deposition, but also offers novel potential targets for genetic improvement of abdominal fat deposition and meat quality in poultry breeding.

## CRediT authorship contribution statement

**Shuohan Li:** Data curation, Investigation, Validation, Writing – original draft. **Xi Cheng:** Formal analysis, Validation, Software. **Ke Zhang:** Software, Visualization, Validation. **Yang Wang:** Visualization, Validation, Investigation. **Hongyu Wei:** Visualization, Validation, Investigation. **Yihao Zhi:** Formal analysis, Software, Visualization. **Zhimin Cheng:** Software. **Yulong Guo:** Supervision. **Hong Li:** Funding acquisition, Methodology, Supervision. **Yadong Tian:** Methodology, Supervision. **Xiaojun Liu:** Conceptualization, Methodology, Resources, Writing – review & editing. **Weihua Tian:** Conceptualization, Funding acquisition, Project administration, Writing – review & editing.

## Disclosures

The authors declare no conflict of interest.

## Appendix Supplementary materials

Image, application 1

Image, application 2
