# Supplementary material for: Genome-wide analysis of TMEM38 family revealed functional roles of TMEM38B in fat deposition and its miRNA-mediated regulation in chicken
Source: Poult Sci. 2025 Aug 16;104(11):105694. doi: 10.1016/j.psj.2025.105694 (PMC12396434; doi:10.1016/j.psj.2025.105694)
Supplement: Supplementary file 1 [file mmc1.docx]

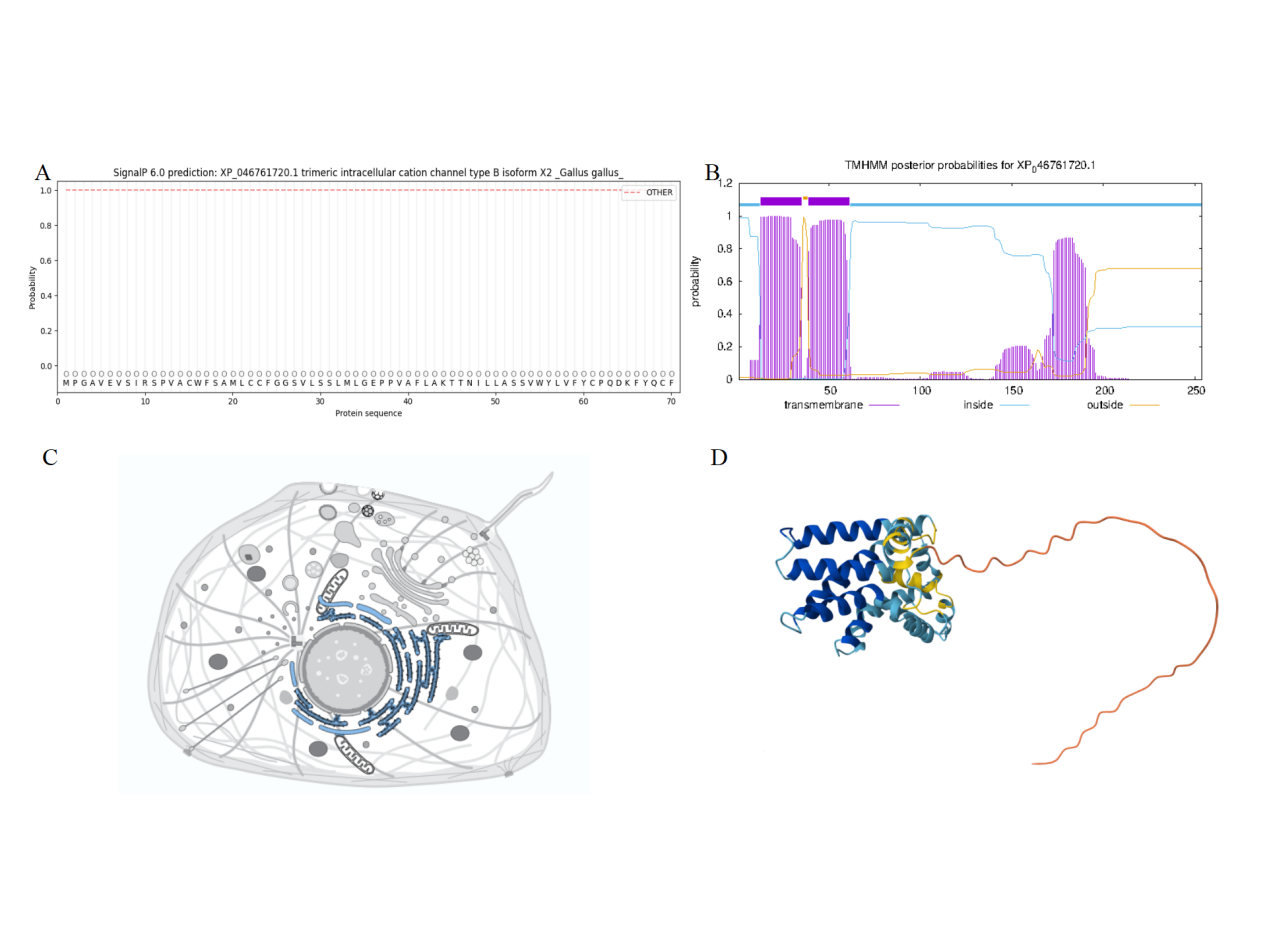


Supplementary Figure S1. Bioinformatics analysis of chicken TMEM38B protein. (A) Signal peptide prediction of chicken TMEM38B protein. (B) Transmembrane structure analysis of chicken TMEM38B protein. (C) Subcellular localization prediction of chicken TMEM38B protein. (D) Secondary structure map of chicken TMEM38B protein.


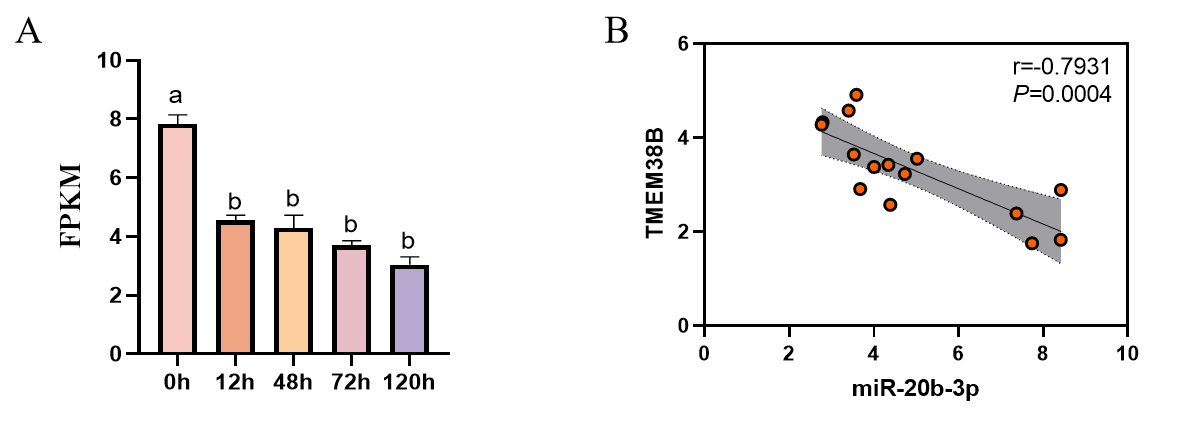


Supplementary Figure S2. (A) miR-20b-3p expression during adipogeneic differentiation of chicken abdominal preadipocytes by RNA-seq. (B) Correlation analysis of *TMEM38B* gene expression and miR-20b-3p expression.
